# Characterization of the Ability of Low-Cost GNSS Receiver to Detect Spoofing Using Clock Bias

**DOI:** 10.3390/s23052735

**Published:** 2023-03-02

**Authors:** Victor Truong, Alexandre Vervisch-Picois, Jose Rubio Hernan, Nel Samama

**Affiliations:** 1 France Développement Conseil (FDC), 10 Cours Louis Lumière, 94300 Vincennes, France; 2 Service réparts, Architecture, Modélisation, Validation, Administration des Réseaux (SAMOVAR), Télécom SudParis, Institut Polytechnique de Paris, 91120 Palaiseau, France

**Keywords:** spoofing, interferences, clock bias, clock drift, GNSS, UAV, GPS

## Abstract

The aim of this paper was to propose a method to characterize the ability of a GNSS user to detect a spoofing attack from the behavior of the clock bias. Spoofing interference is not a new issue, especially in military GNSS, although it is a new challenge for civil GNSS, since it is currently implemented and used in many everyday applications. For this reason, it is still a topical issue, especially for receivers that only have access to high-level data (PVT,CN0). To address this important issue, after conducting a study of the receiver clock polarization calculation process, this led to the development of a very basic Matlab model that emulates a spoofing attack at the computational level. Using this model, we were able to observe that the clock bias is affected by the attack. However, the amplitude of this disturbance depends on two factors: the distance between the spoofer and the target and the synchronization between the clock that generates the spoofing signal and the reference clock of the constellation. To validate this observation, more or less synchronized spoofing attacks were carried out on a fixed commercial GNSS receiver with the use of GNSS signal simulators and also with a moving target. We propose then a method to characterize the capacity of detecting a spoofing attack with the clock bias behavior. We present the application of this method for two commercial receivers of the same manufacturer from different generations.

## 1. Introduction

Global Navigation Satellite System (GNSS) receivers are the primary technology used for positioning and navigation. The popularity of GNSS, especially the Global Positioning System (GPS) and GLObal NAvigation Satellite System (GLONASS), comes from its open-access standards [1] and its ease of use. Besides, cost reductions and miniaturization of the electronics helped increase its popularity. This is why systems such as Unmanned Aerial Vehicles (UAVs) rely heavily on GNSS receivers for navigation and positioning. However, civil GNSS receivers are often exposed to different kinds of interference, which can be intentional [1]. Such malicious interferences are a great danger for systems that are highly reliant on GNSS during their functioning. One of these malicious interferences is spoofing, which consists of broadcasting counterfeit GNSS signals in order to confuse the targeted receiver about its real position. It is worth noting that a spoofer can manipulate the trajectory of a UAV.

The remaining sections are structured as follows. Section 2 focuses on the background of GNSS spoofing. Section 3 focuses on the computation of the clock bias and the functioning of a GNSS receiver’s clock. This section also investigates the influence of a spoofer on the clock bias. Section 4 details several experiments on the effects of spoofing on the clock of a real commercial GNSS receiver. Section 5 presents a method to characterize the capacity of detecting a spoofing attack on a commercial receiver. Section 6 discusses the approach analyzed in the paper. Finally, Section 7 concludes the paper.

## 2. GNSS Spoofing and Detection Techniques

### 2.1. GNSS Spoofing

Spoofing consists of broadcasting non-legitimate GNSS signals with the intention that the receiver calculates a false position, i.e., a position different from the real position of the target. As explained in [2], spoofers can be classified on the hardware level into three categories:

1. The “simple” GNSS signal simulators.
2. The receiver-based spoofers.
3. The sophisticated receiver-based spoofers.

The first category includes GNSS simulators mimicking authentic GNSS signals. Even though these signals are noise to the targeted receiver, this is sufficient to mislead a commercial GNSS receiver. The receiver-based spoofers are spoofers that combine a GNSS signal simulator and a GNSS receiver. The receiver part allows the spoofer to synchronize its signals to the current GNSS signal so that the fake signals can easily mislead the target. The third category covers receiver-based spoofers that can perfectly synchronize their signals with the authentic ones; this also covers spoofers using several antennas mimicking a GNSS constellation.

In order to understand how a spoofer can mislead a receiver, it is necessary to look at the signal model. As stated in [3], a GNSS signal can be mathematically expressed by

(1) $$y\left(t\right)=Re\left\{\sum_{i=1}^{N}A_{i}D_{i}\left[t-\tau_{i}\left(t\right)\right]C_{i}\left[t-\tau_{i}\left(t\right)\right]e^{j[\omega_{c}t-\phi_{i}\left(t\right)]}\right\}$$

with N the number of signals and $A_{i}$, $D_{i}$, $\tau_{i}$, $C_{i}$, $\omega_{c}$, and $\phi_{i}$, respectively, the carrier amplitude, the data bits, the code phase, the spreading code, the carrier frequency, and the carrier phase of the ith signal. Spoofing signals must have the same spreading codes as the true signals and a good estimate of their data bits in order to deceive a receiver. This leads to three metrics that the spoofer can work with to perform its attack. As emphasized by Broumandan et al. [4], these metrics are the relative power ($A_{i}$), the relative delay ($\tau_{i}$), and the relative phase ($\phi_{i}$). The way the spoofer will exploit them will define which type of spoofing attack will be used. Thus, the spoofing signal can be expressed as follows:

(2) $$y_{s}\left(t\right)=Re\left\{\sum_{i=1}^{N}A_{si}\hat{D_{i}}\left[t-\tau_{si}\left(t\right)\right]C_{i}\left[t-\tau_{si}\left(t\right)\right]e^{j[\omega_{c}t-\phi_{si}\left(t\right)]}\right\}$$

A self-consistent spoofer is a type of spoofer that uses these metrics without any significant impact on pseudorange residuals [3]. The main challenge of these spoofers, besides defeating Receiver Autonomous Integrity Monitoring (RAIM) focusing on pseudorange residuals, is to make the target lock onto the fake signals. There are two main ways to do this: (i) Jamming the target to force the loss of the legitimate signal, returning the receiver’s GNSS algorithm to acquisition mode. Later, broadcast spoofed signals with a power level $A_{si}$ higher than legitimate signal power $A_{i}$ ($A_{si}\gg A_{i}$). (ii) Broadcasting fake signals, matching their code phase and carrier phase to those of the legitimate signals at the target location (i.e., $\tau_{si}\approx \tau_{i}$) starting at a low power ($A_{si}\approx 0$). Then, $A_{si}$ is increased until $A_{si}>A_{i}$ before shifting $\tau_{si}$ away from $\tau_{i}$ to drag the victim to the desired position. This method needs the spreading code and the data bits to be predictable to set them correctly on the spoofing signals. When that is not the case, a spoofer can perform another form of spoofing attack, which is called meaconing. This consists of recording the true GNSS signals and replaying them with an added time delay. Self-consistent spoofers have been implemented and tested with success by Shepard et al. [5] and Humphreys et al. [6]. Those papers showed that it is possible to build a relatively low-cost GPS spoofer using Commercial-Off-The-Shelf (COTS) components. That explains why a self-consistent spoofer is the most-common type of spoofer.

Besides these techniques, there are more advanced forms of spoofing. One of them, called *nulling*, consists of transmitting two types of signals, the spoofed signals that induce a fake position and the nulling signals that “cancel” all the true signals. Another advanced spoofing technique is the use of multiple spoofers and/or multiple antennas. However, this kind of spoofing method is very difficult to implement.

### 2.2. Spoofing Detection Techniques

An overview of the previous work performed on spoofing detection techniques led by Psiaki et al. [3] showed that the detection techniques can be classified into five groups (signal processing, encryption, geometric, drift monitoring, and mixed techniques):

- **Signal-processing-based techniques** focus on the signal characteristics to detect abnormal behavior or distortions. In [4], different detection metrics used in signal processing techniques were listed. Most techniques monitor the received power, since a basic spoofing attack adds signals, which will increase the received power. Akos et al. [7] showed that a spoofing attack causes high variations of the gain level of the receiver’s Automatic Gain Control (AGC). Hence, monitoring the AGC seems to be a good idea to detect GNSS spoofing. However, focusing only on received power does not allow the detection of more subtle spoofing attacks, such as a spoofer using low-power signals. Monitoring both the received power and the distortions of the correlation function can counter this issue. A version of a “Power-Distortion” (PD) detector in [8] showed good results at detecting GNSS interferences and can also be used to classify them. This last feature was improved in [9] by using maximum likelihood estimation as a distortion detector instead of the symmetric difference.
- **Encryption-based techniques** are mainly based on authentication. Curran et al. [10] carried out an overview of the coding schemes in GNSS in order to highlight that the use of authentication codes in the GNSS signals could increase the system integrity. An interesting approach suggested by Psiaki et al. [11] exploits the similarities between the civil and military GPS signals to detect spoofing. However, this kind of solution is not easy to implement.
- **Geometry-based detection techniques** look for the direction of arrival of the signals. This can be achieved by using multiple antennas and/or multiple receivers. Montgomery et al. [12] used two antennas with a single receiver to calculate the angle of arrival of the signals, but this required dedicated hardware. A similar approach was successfully implemented with Commercial-Off-The-Shelf (COTS) components in [13,14], where two COTS receivers with one antenna each were used.
- **Drift-monitoring techniques** mainly focus on unexpected changes of other metrics available on a GNSS receiver, which are the clock or the position. Marnach et al. [15] focused on the clock bias of a GPS receiver to detect meaconing (a simplified form of spoofing) attacks, and Liu et al. [16] worked on the influence of spoofing attacks on the pseudo-ranges and the clock bias.
- **Mixed techniques** consist of combining two or more techniques from the groups described previously to complement each other. Mixing GNSS-based detection techniques with other information coming from other sensors also belongs in this group. The most-common idea is to use an Inertial Measurement Unit (IMU) to exploit inertial measurements to compare with the positioning given by the GNSS data. Zou et al. [17] modeled the behavior of a UAV and compared it to the real positioning information coming from the IMU and the GPS receiver. Panice et al. [18] and Tanil et al. [19] used data fusion between the IMU and GPS and focused on the detection of the abnormal results of the data fusion to detect spoofing attacks.

This background work shows that self-consistent spoofers are not a negligible threat. Many research works on spoofing detection focused on signal processing or geometric properties in order to counter that kind of spoofer. However, implementing those techniques can require additional hardware or more computational resources. On the other hand, drift-monitoring techniques can be implemented on some systems with the hardware already installed, such as UAVs for example, but these types of spoofing detection techniques have not been thoroughly investigated. Marnach et al. [15] worked on this, but only with meaconing, which is not as complex or deceitful as spoofing. Lui et al. [16] also looked at that lead, but they mainly focused on the pseudo-range of the SVs. This paper focused on drift-monitoring techniques by investigating the effects that a spoofing attack will induce on the clock bias and the clock drift of a GNSS receiver. The advantage of monitoring the clock bias and the clock drift of a receiver is that these high-level data are relatively easy to obtain on most receivers. According to leading experts in the field, for commercial receivers, the time variable, thus the clock bias, is the most-relevant for detecting spoofing [3]. It is interesting to notice that works on the use of clock bias to detect spoofing have already been carried out [20]. The claimed originality of our work consists of proposing a method to characterize the capacity of a commercial receiver to detect spoofing attacks of the three categories mentioned above, mainly with clock bias observation.

## 3. GNSS Receiver’s Clock

### 3.1. Computation of a GNSS Position

GNSS positioning is based on Time Of Arrival (TOA) ranging, as explained in [1,21]. TOA ranging consists of calculating the propagation time of a signal to calculate the distance between the sender of the signal and the receiver. To compute the position of a user, the receiver has to measure the “pseudo-range” between the user and the satellites—Space Vehicles (SVs)—of a GNSS constellation. This measure represents not only the range between the SVs and the receiver, but also the time difference between the SVs and the receiver clocks, hence the word “pseudo”. Considering that the positions of the SVs are known, thanks to the ephemerides data, it is possible to deduce the position of the user, solving the following system of equations:

(3) $$\rho_{j}=\sqrt{{\left(x_{j}-x_{u}\right)}^{2}+{\left(y_{j}-y_{u}\right)}^{2}+{\left(z_{j}-z_{u}\right)}^{2}}+ct_{u}$$

with *j* referring to the satellites, $\rho_{j}$ and $\left(x_{j},y_{j},z_{j}\right)$, respectively, the so-called pseudo-range and the coordinates of the satellite *j*, $\left(x_{u},y_{u},z_{u}\right)$ the coordinates of the user in the Earth-Centered Earth-Fixed (ECEF) system, *c* the speed of light, and $t_{u}$ the receiver’s clock bias. In order to compute the receiver’s position and the clock bias, at least four equations (four visible SVs) are needed to solve the system of equations, which means that *j* ranges from 1 to 4 at least. As explained in [1], one way of solving that system is to use iterative techniques based on linearization. With four equations, the solution is $\Delta x=H^{-1}\Delta \rho$, with *H* the matrix of the linearized form of the system (3), whose expression is detailed in Section 3.3. If the system is over-determined, the solution of the system can be approximated with the least-squares method:

(4) $$\Delta \tilde{x}={\left(H^{T}H\right)}^{-1}H^{T}\Delta \rho$$

### 3.2. Clock Bias Computation and Usual Behavior

The clock bias represents the time difference between the clock of the satellites of a GNSS constellation and the receiver’s clock. The clocks of the satellites are atomic clocks with a stable drift, while the clocks of the receivers are generally implemented using a quartz crystal, sometimes compensated in temperature (TCXO), whose stability cannot compete with atomic clocks. As explained in [21], the satellite and receiver synchronization problems are very different. The satellite synchronization problem is solved from the *control segment* and using specific computation such as Kalman filtering [1]. Otherwise, it is solved with the system of Equation (3). Let N be the number of SVs used and $h_{4,j}$ the *j*th term of the last row of the matrix of the solution (i.e., $H^{-1}$, if N=4 and ${\left(H^{T}H\right)}^{-1}H^{T}$, if N>4). Then, the clock bias term of the system of equations can be expressed as:

(5) $$\Delta t_{u}=-\frac{1}{c}\sum_{j=1}^{N}h_{4,j}\Delta \rho_{j}$$

**Figure 1:**
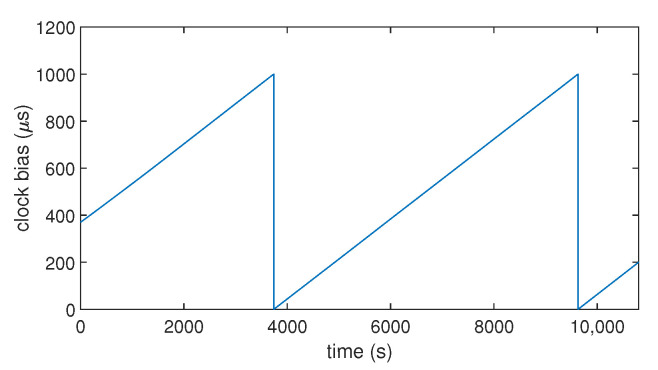
Clock bias over time computed by a commercial GNSS receiver (u-blox 8T).

**Figure 2:**
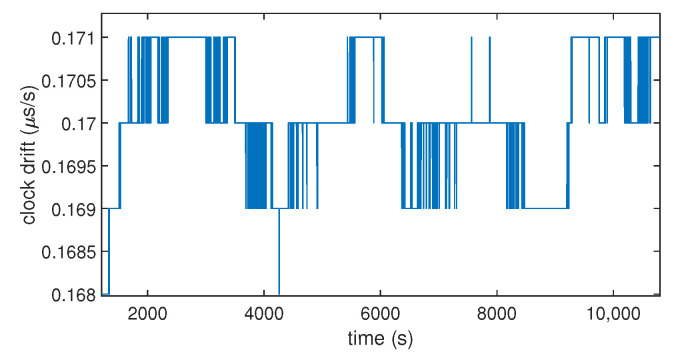
Clock drift over time computed by a commercial GNSS receiver (u-blox 8T).

To understand how the clock bias of a commercial low-cost GNSS receiver behaves in normal conditions, we recorded the clock bias of a commercial low-cost GPS receiver over 3 h (10,800 s), as shown in Figure 1. As expected, we found that the clock bias has a stable variation over time. Its evolution over time is similar to a saw-tooth signal.This comes from the fact that the receiver resets its clock bias when it reaches 1 ms. All receivers do not work like this (it could be ±0.5 ms according to the settings). Moreover, there is another important phenomenon influencing the time synchronization: the clock drift. It is the first derivative over time of the clock bias. Although the evolution of the clock bias seems linear (cf. Figure 1), Figure 2 shows that the drift has slight variations of its value over time.

### 3.3. Spoofer Influence on the Clock Bias

A spoofer will transmit false signals, which will affect the system of equations by adding “error” terms to the satellite position and pseudo-range. Let $\epsilon_{\rho }$, $\epsilon_{x}$, $\epsilon_{y}$, and $\epsilon_{z}$ be those terms. Thus, Equation (3) can be expressed during a spoofing attack as

(6) $$\rho_{j}^{\prime }=\sqrt{{\left(x_{j}^{\prime }-x_{u}\right)}^{2}+{\left(y_{j}^{\prime }-y_{u}\right)}^{2}+{\left(z_{j}^{\prime }-z_{u}\right)}^{2}}+ct_{u}$$

where:

(7) $$\left\{\begin{array}{cc} \rho_{j}^{\prime } & =\rho_{j}+\epsilon_{\rho }\\ x_{j}^{\prime } & =x_{j}+\epsilon_{x}\\ y_{j}^{\prime } & =y_{j}+\epsilon_{y}\\ z_{j}^{\prime } & =z_{j}+\epsilon_{z} \end{array}\right.$$

The linearized form of Equation (6) is then

(8) $$\Delta \rho_{j}^{\prime }=a_{x_{j}}\Delta x_{u}+a_{y_{j}}\Delta y_{u}+a_{z_{j}}\Delta z_{u}-c\Delta t_{u}$$

$$\mathrm{where}\;\left\{\begin{array}{c} a_{x_{j}}=\frac{x_{j}^{\prime }-\hat{x_{u}}}{\hat{r_{j}}},\;a_{y_{j}}=\frac{y_{j}^{\prime }-\hat{y_{u}}}{\hat{r_{j}}},\;a_{z_{j}}=\frac{z_{j}^{\prime }-\hat{z_{u}}}{\hat{r_{j}}}\\ \hat{r_{j}}=\sqrt{{\left(x_{j}^{\prime }-\hat{x_{u}}\right)}^{2}+{\left(y_{j}^{\prime }-\hat{y_{u}}\right)}^{2}+{\left(z_{j}^{\prime }-\hat{z_{u}}\right)}^{2}}\\ \Delta x_{u}=x_{u}-\hat{x_{u}},\;\Delta y_{u}=y_{u}-\hat{y_{u}},\;\Delta z_{u}=z_{u}-\hat{z_{u}}\\ \Delta t_{u}=t_{u}-\hat{t_{u}},\;\Delta \rho_{j}^{\prime }=\hat{\rho_{j}^{\prime }}-\rho_{j}^{\prime } \end{array}\right.$$

The system of linearized equations can be expressed in matrix form Δρ=HΔX, with

$$\begin{array}{c} \Delta \rho =\left[\begin{array}{c} \Delta \rho_{1}\\ \vdots \\ \Delta \rho_{j}\\ \vdots \\ \Delta \rho_{N} \end{array}\right],\;H=\left[\begin{array}{cccc} a_{x_{1}} & a_{y_{1}} & a_{z_{1}} & 1\\ \vdots & \vdots & \vdots & \vdots \\ a_{x_{j}} & a_{y_{j}} & a_{z_{j}} & 1\\ \vdots & \vdots & \vdots & \vdots \\ a_{x_{N}} & a_{y_{N}} & a_{z_{N}} & 1 \end{array}\right],\;\\ \mathrm{and}\;\Delta X=\left[\begin{array}{c} \Delta x_{u}\\ \Delta y_{u}\\ \Delta z_{u}\\ -c\Delta t_{u} \end{array}\right] \end{array}$$

When N=4, the solution is

$$\left[\begin{array}{c} \Delta x_{u}\\ \Delta y_{u}\\ \Delta z_{u}\\ -c\Delta t_{u} \end{array}\right]={\left[\begin{array}{cccc} a_{x_{1}} & a_{y_{1}} & a_{z_{1}} & 1\\ a_{x_{2}} & a_{y_{2}} & a_{z_{2}} & 1\\ a_{x_{3}} & a_{y_{3}} & a_{z_{3}} & 1\\ a_{x_{4}} & a_{y_{4}} & a_{z_{4}} & 1 \end{array}\right]}^{-1}\left[\begin{array}{c} \Delta \rho_{1}\\ \Delta \rho_{2}\\ \Delta \rho_{3}\\ \Delta \rho_{4} \end{array}\right]$$

Therefore, after the linearization of the system, the error terms are still present in the matrix *H*. Equation (5) can be applied here with $h_{4,j}$ being the term of the last row of $H^{-1}$, which will contain the error terms introduced by the spoofer. This shows that a spoofing attack will likely impact the value of the clock bias. Because of the size of the matrix, *H*, it does not easily give a clear analytical expression of the clock bias under a spoofing attack, especially when N>4. Hence, a numerical model was implemented in Matlab in order to characterize the influence of the spoofer on the clock bias of a GNSS receiver.

### 3.4. Simplified Model Approach

To show the influence of a spoofing attack on the clock bias, a simple Matlab model was developed to emulate a self-consistent receiver spoofing attack. The model emulates the spoofing attack only on the computation level of the receiver, which means that only the equations used to compute a GPS position are taken into account, not the signal model nor the power level. The chosen spoofing attack for this model is a simple one. It is an attack that will induce a fixed fake position (i.e., the position that the spoofer wants the receiver to calculate, instead of the real one) on a fixed GPS receiver. The spoofer is supposed to be “perfect”, i.e., the spoofing constellation is identical to the real one (the same SVs, the same positions, and the same time). It is worth noting that, in the results shown hereinafter, the fake position matched the location of the spoofer, being 240 m away from the real position.

**Figure 3:**
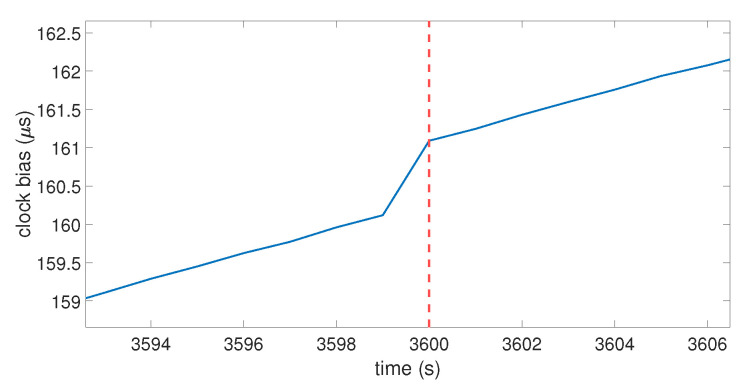
Clock bias over time on the model. The red dashed line indicate the moment that the receiver switches to the fake position calculation (spoofing constellation).

**Figure 4:**
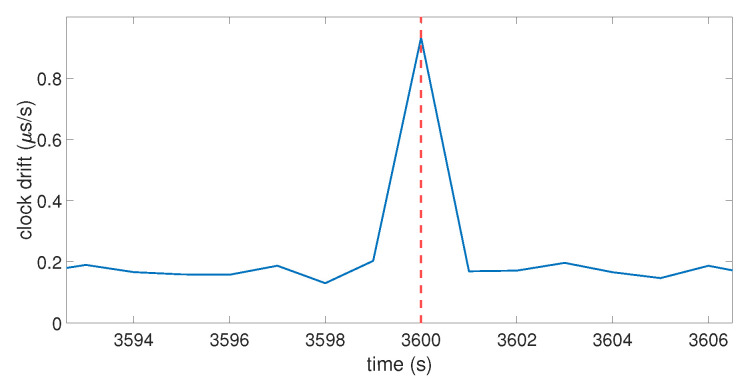
Clock drift over time on the model. The red dashed line indicate the moment that the receiver switches to the fake position calculation (spoofing constellation).

The code emulates the clock bias of a GPS receiver for an arbitrary duration by using a time loop. The clock bias behaves like the one shown in Figure 1 by setting an initial drift with an initial value for the bias. The SVs were emulated through their positions thanks to the use of real ephemerides data. The same ephemerides were used to emulate two perfectly synchronized GPS constellations: one for the real signals, and the other one for the spoofer. This allowed the calculation of the pseudo-ranges corresponding to the desired positions. A noise term was also used to emulate the natural noise that occurs in pseudo-range data. At each second, the positions of the SVs were updated, and the GPS position computation process based on the equations described in Section 3.1 was launched to obtain the coordinates and the clock bias of the receiver at that time. At an arbitrary moment of the timeline, the receiver was forced to switch from the real constellation to the spoofing constellation. Let $t_{s}$ be that moment. Figure 3 and Figure 4 show the clock bias and the clock drift computed by the emulated GPS receiver.

In this case, $t_{s}=3600$ s, which is highlighted by the vertical red dotted line. For $t<t_{s}$, the real pseudo-ranges were used in the position computation process; for $t\geq t_{s}$, the fake pseudo-ranges (i.e., $\rho_{j}^{\prime }$ in Equation (7)) were used instead. The spoofing pseudo-ranges were set as the addition of the pseudo-ranges between the SVs and the fake point and the distance between the targeted receiver and the spoofer’s antenna. Figure 3 shows that, when the receiver switches to the spoofing constellation, the clock bias has a jump in value. This jump translates in Figure 4 into a peak in the clock drift at the same moment. The amplitude of the clock bias leap and the clock drift peak are not constant over multiple iterations of the same spoofing attack, due to the random noise term used in the pseudo-ranges. Globally, the clock bias jump is about 1 μs and the clock drift peak is about 0.9 μs/s. The model shows that a spoofing attack on a GNSS receiver will cause a leap of value on its clock bias, which will also affect the clock drift. To investigate if a spoofer can mitigate the amplitude of this jump of value, the influence of different parameters of the model is tested in the next section.

### 3.5. Influence of the Different Parameters

**Figure 5:**
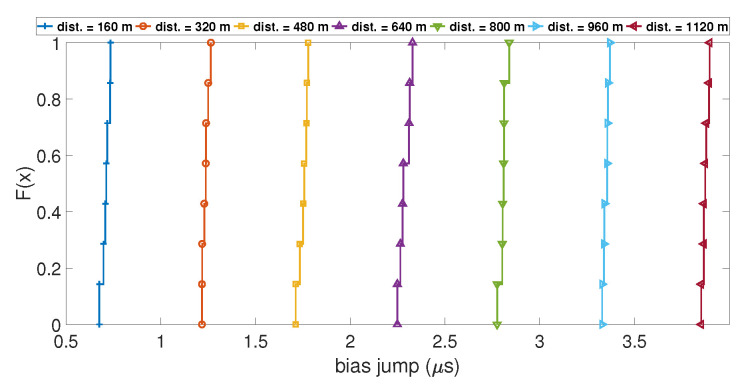
ECDF of the clock bias jump for different distances.

**Figure 6:**
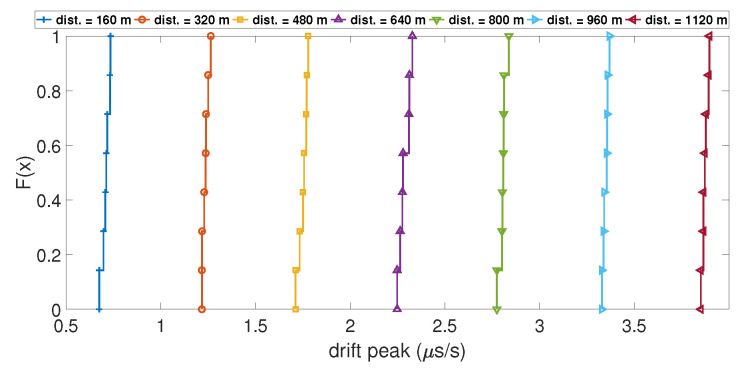
ECDF of the clock drift peak for different distances.

In order to understand which parameters of the model will likely increase the value of the clock bias jump during a spoofing attack, the variation of four parameters was tested. These parameters were: (i) the number of SVs used during the computation; (ii) the initial drift used to model the clock bias; (iii) the distance between the target and the spoofing antenna; and (iv) the time de-synchronization between the two GPS constellations. To show their influence, the Empirical Cumulative Distribution Function (ECDF) of the value of the clock bias jump and the ECDF of the amplitude of the peak of the drift were calculated for different values of the parameter of interest. An ECDF is plotted with a dataset of measured clock bias jumps and clock drift peaks during 100 iterations of the Matlab model. The ordinate of the plot F(x) is the percentage of data from the dataset that is less than or equal to the value *x*. If ECDFs are computed for different values of a parameter, it is interesting to compare them with each other. Figure 5 shows the ECDF of the clock bias jump for different distances between the receiver and the spoofing antenna. The ECDFs for each distance are well separated, and the ECDF of the smallest distances shows a smaller value of clock bias jump than the ECDF in the higher distances. This is the same case with the ECDF of the clock drift peak, as shown in Figure 6. This highlights that the distance between the targeted receiver and the spoofing antenna has a clear influence on the amplitude of the clock bias jump caused by a spoofing attack: the higher the distance, the higher the amplitude of the jump is.

**Figure 7:**
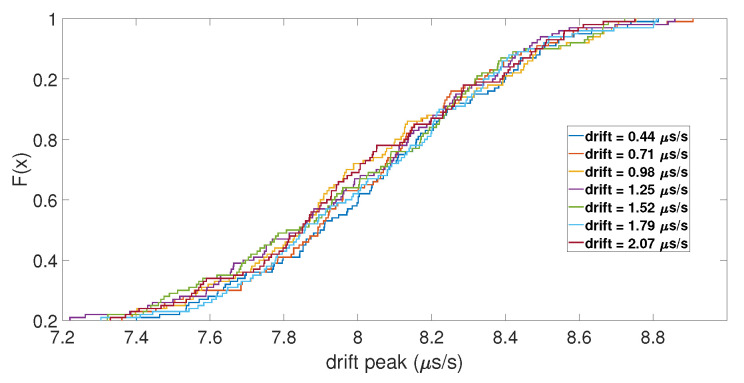
ECDF of the clock drift peak for different initial drifts.

**Figure 8:**
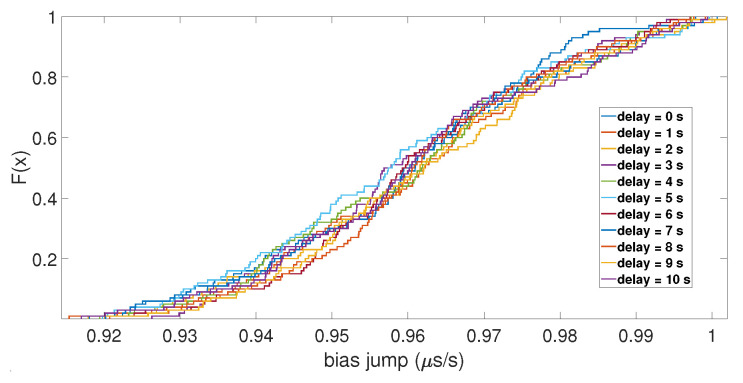
ECDF of the clock bias jump for different delays between the two constellations.

With the initial drift, the same results were observed for the value of the clock bias jump, but not for the amplitude of the clock drift peak. As shown in Figure 7, the ECDFs of the drift peak were mixed together, which indicates that the initial drift did not impact the peak of the clock drift caused by the spoofing attack. Regarding the number of SVs used for the computation, the ECDFs showed that it had no impact. Likewise, for the de-synchronization between the two constellations, it seemed that it did not have an influence on the clock bias jump and the amplitude of its drift, as shown in Figure 8.

Therefore, over the parameters tested, the distance, between the target and the spoofer, was the one that had the most impact on the amplitude of the clock bias jump and the clock drift peak. Now that the effects of a spoofing attack on the clock of the receiver have been highlighted by our model, it is necessary to see if the same phenomenon happens on a commercial GNSS receiver and if the amplitudes of these effects are similar (cf. Section 4).

## 4. Spoofing Experiments on a Commercial GNSS Receiver and Detection Characterization

If we look at the previous model and the presented results, we will have understood that we expect behavior that is fairly easy to predict. To be efficient, the clock bias approach requires a large “distance” between the receiver and the spoofer. This “distance” could also be a consequence of the change of the reference clock (from the real constellation to the spoofer constellation). At this stage, it seems necessary to carry out some experimentations with a commercial receiver and check the expected behaviors.

### 4.1. Fixed Spoofing Attack Using Two Different Clocks

**Figure 9:**
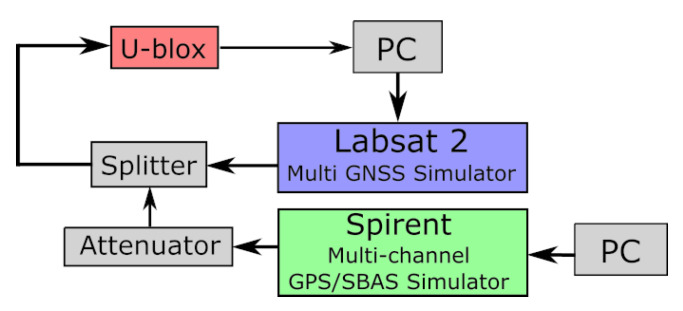
Experimental setup. The black arrows represent the wired link. The splitter and attenuator are radio frequency components.

To investigate the effects of a spoofing attack, it is necessary to carry out a spoofing attack with a GNSS signal generator that will broadcast the spoofing signals. However, due to legal reasons, it is not possible to set up such an experiment. Therefore, all our experiments were implemented using wired hardware, as shown in Figure 9. To carry out the experiments, two GNSS signal simulator were used: the LabSat 2 and the Spirent GSS6560. The LabSat is a GNSS signal simulator that can record and repeat GNSS signals, whereas the Spirent is a GPS signal simulator that can simulate a complete GPS constellation on a maximum of 12 channels and offers many parameters to configure. The GNSS receiver is a receiver manufactured by u-blox that is delivered with software that allows the user to collect a large amount of data from the receiver on a computer. Two models of the u-blox were tested: u-blox 6T (u-blox chipset Version 6 with Precision Timing) and u-blox M8T (u-blox chipset Version M8 with Precision Timing), hereinafter referred to as u-blox 6 and u-blox 8. Figure 9 shows the experimental setup.

**Figure 10:**
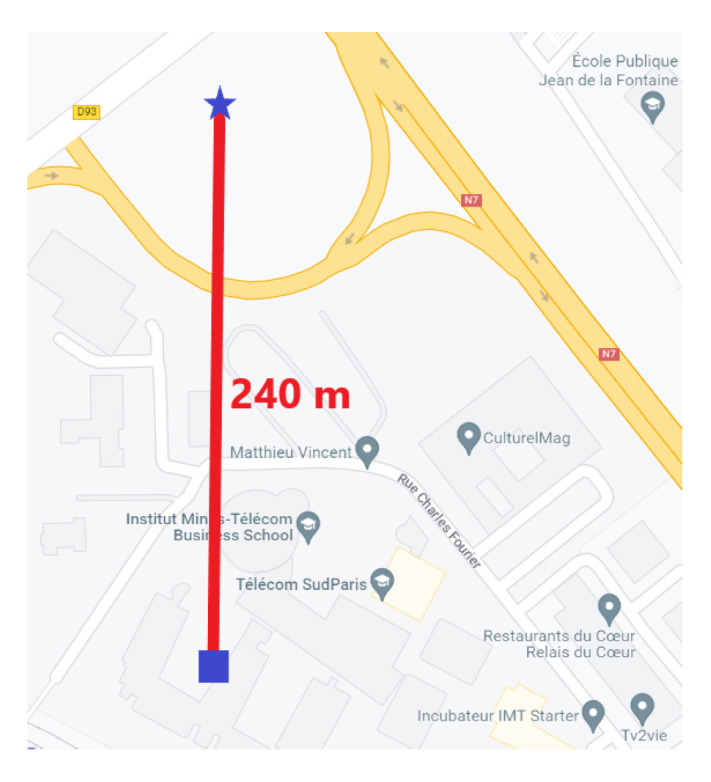
The true position corresponding to the “real” signals and the “fake” one corresponding to the spoofer signal.

The attack scenarios focused on the GPS signals. The position considered was static, and the signal conditions of reception were the standard ones: Without the spoofing attack, we had 10 satellites in visibility with 38 to 48 dBHz CN0 (according to the receiver), depending on the satellites elevations. The signal of the spoofer corresponds to the same constellation, but was located at another position 240 m away. Figure 10 shows the relative position on the map.

During these experiments, the LabSat was used in addition to the Spirent. Since the LabSat is a repeater, the Spirent simulated the spoofer, while the LabSat simulated the True Satellites in Visibility (TSVs) by repeating a recording of the GNSS signals previously simulated by the Spirent.

To carry out the attack, we considered two ways:

- The Larger Power (LP) of the spoofer compared to the “true” signals.
- The Lost True Signal (LTS) to force the receiver to relaunch the signal acquisition process.

Four scenarios of the spoofing attack were tested:

- Smooth attack: starts with low-power signals (−65 dBm on the Spirent), which are gradually increased (5 dB by 5 dB) until the target is spoofed.
- Strong attack: starts with high-power signals (−40 dBm on the Spirent).
- Jamming attack: starts with a high-power noise to jam the target before broadcasting the spoof signals (−65 dBm on the Spirent).
- Acquisition mode: similar to the smooth attack, but the receiver is forced into acquisition mode at each rise in power (5 dB by 5 dB).

These attacks can be considered of the first category defined in Section 2.1. No synchronization was realized between the LabSat (real constellation) and SPIRENT (spoofer constellation), which were launched separately “by hand”.This may seem trivial at first glance, but perfect synchronization of the attacker with the real constellation is neither obvious nor easy to achieve. An example of successfully conducted spoofing experiments to trick the Google map software was given [22] without any kind of synchronization. There was only a Software-Defined Receiver, which replayed the spoofing signal.

**Figure 11:**
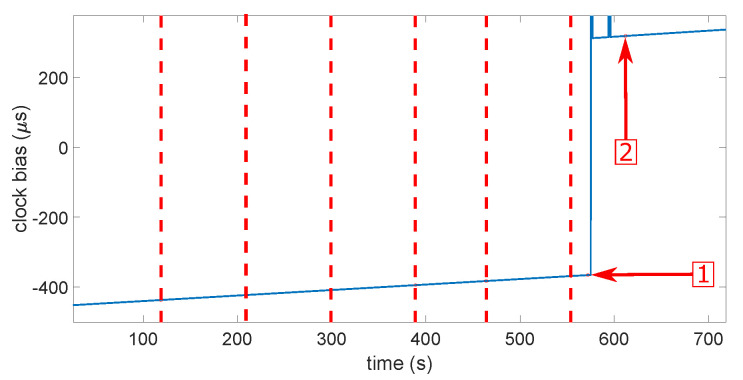
Clock bias during a smooth attack. The red dashed lines indicate each power increase on the Spirent. The first arrow shows the moment when the receiver loses the “real” constellation. The second arrow shows the moment when the receiver locks onto the spoofer.

**Figure 12:**
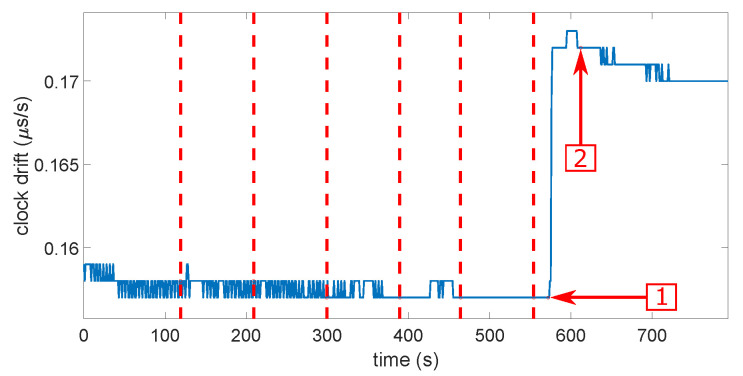
Clock drift during a smooth attack. The red dashed lines indicate each power increase on the Spirent. The first arrow shows the moment when the receiver loses the “real” constellation. The second arrow shows the moment when the receiver locks onto the spoofer.

Then, it does not seem so simple to determine what would be the most-realistic scenarios in a real case of a spoofing attack. Probably the three firsts, but the most-important is that each scenario be tested several times with success. Figure 11 and Figure 12 show the clock bias and its drift during a smooth attack. The red dashed lines indicate each power increase on the Spirent. The first arrow shows the moment when the receiver loses the “real” constellation. The second arrow shows the moment when the receiver locks onto the spoofer. We can see that both the clock bias and its drift showed an abnormal leap of value when the receiver switched from the real signals to the spoofing signals. The same behavior was noticed on the other types of spoofing attack on both receivers, but in some cases with the u-blox 6, the clock drift showed no jump. This led to the first limitation of the approach. Theoretically, we should observe a jump, but there was not one. The computation of the clock drift probably has a smoothing process that we do not know. This means that, if we want to characterize the clock bias approach, it appears difficult not to do this empirically. That sounds quite logical. Indeed, the processes for calculating clock bias and drift do not take into account the possibility of a spoofing attack. They were obtained with as much stability as possible to be provided to the user. They were, therefore, calculated by smoothing out the variations, probably under the effect of a Kalman filter or something equivalent. We will confirm this observation with more details in the next section during the characterization.

Table 1 sums up the mean values of leap measured with all the types of attack, with the Carrier-to-Noise (C/No) ratio (which is the signal-to-noise ratio on a 1 Hz bandwidth) measured on the u-blox in the case of a successful spoofing attack. It is worth noting that Table 1 only shows mean values, but during our tests, the clock bias jump ranged from 100 μs to a little more than 1 ms, while the clock drift jump did not present these deviations. These experiments confirmed our previous observations regarding the effects of a spoofing attack. The large order of magnitude of the clock bias jump can be explained by the fact that the two constellations were simulated with two independent GPS signal simulators. This led to a time delay between the clocks of the two constellations. There was also a difference in the specifications of their clocks.

To highlight the randomness of the clock bias jump and to evaluate its amplitude, the spoofing attack was automatically repeated with many successive iterations. Thus, we will see if the amplitude of the clock bias jump has a certain consistency among those iterations.

### 4.2. Results of Successive Iterations of a Fixed Spoofing Attack

**Figure 13:**
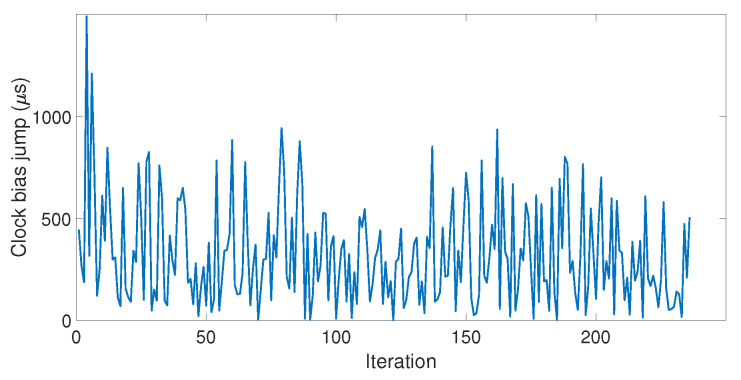
Clock bias jump measured at each iteration.

Since the LabSat and the Spirent cannot communicate with each other, each simulator was automatized separately. The LabSat allows the user to choose the delay between each iteration, but the Spirent does not, so the LabSat had to be set so that they both started their iterations at the same time. This caused a time delay between the two simulators, which increased over the iterations. Three datasets of respectively 170, 192, and 256 iterations were carried out (hereinafter referred to as Datasets 1, 2, and 3). Figure 13 shows the value of the clock bias jump measured for each iteration of Dataset 3.

**Figure 14:**
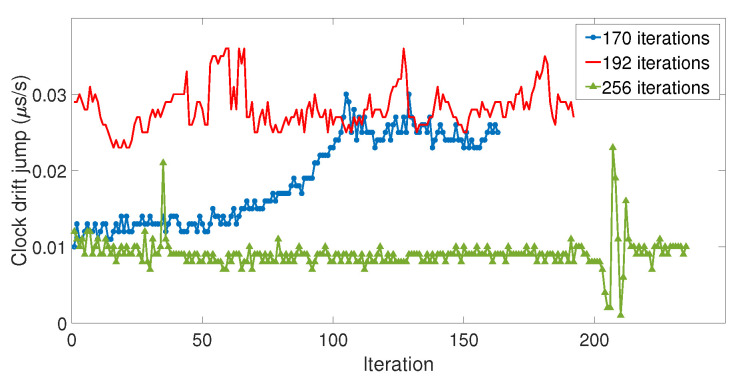
Clock drift jump measured at each iteration.

Among the three datasets, the amplitude of clock bias jump ranged from 0.04 μs to 1.63 ms, with a good proportion of those values being quite high (95% of them were higher than 25 μs). In addition, the amplitude of the clock bias jump did not show consistency over the iterations, which indicated that the amplitude of the clock bias jump was random. Our model did not take into account this randomness, and that is why the amplitude of the clock jump of the model was smaller. Therefore, the spoofing attack caused abnormal leaps of the value on the clock bias whose amplitude could not be predicted due to a random phenomenon. However, this phenomenon is an advantage for the detection: the larger the peak is, the easier the detection. Figure 14 shows the value of the clock drift jump measured for each iteration of the three datasets.

Regarding the clock drift leap of value, its behavior over the iterations was different depending on the series. On the first dataset, the amplitude of the clock drift jump gradually increased over time, whereas on the other series, the fluctuation had no relevant consequences. Besides their length, the difference between the three series was the “speed”of the increase of the delay between the LabSat and the Spirent, which came from the adjustments of the repetition delay of the LabSat. Dataset 1 was the one with the highest “speed” of increase, which can explain the difference with the other datasets. However, considering the amplitude of these jumps, 0.01 to 0.03 μs/s, a 1 to 3 m/s distance equivalent, it seems difficult to use the variation of clock drift to detect the spoofing attack with these receivers.

After these experiments, it appeared that it was possible to use the clock bias against non-synchronized attacks. However, what happens in the case of a synchronized attack? The next section deals with this issue.

## 5. Single Clock Attack and Characterization Method

In this section, we wanted to check the behavior of the previous receivers in the face of a spoofing attack for which the spoofer is synchronized on the real constellation. This led to the following experimentation.

### 5.1. Fixed Spoofing Attack Using a Single Clock

The objective of this experiment was to replicate the same type of spoofing attack as the model of Section 3.4. For this, we generated two sets of signals with the same clock, i.e., with the same GNSS signal simulator, making them perfectly synchronized. As it is difficult to synchronize the signal generation of the LabSat and the SPIRENT, the easiest way to achieve this seems to be to use the same generator of both real and spoofer signals. The GNSS simulator used for this experiment was the Spirent. The drawback of this Spirent generator model is that there is only one Radio Frequency (RF) output, so it cannot output two constellations simultaneously.

**Figure 15:**
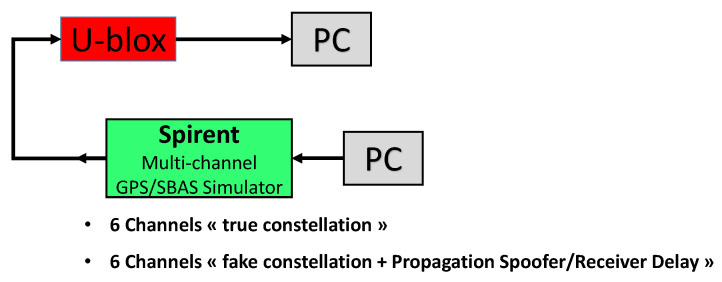
Experimental setup for synchronized constellation spoofing.

Fortunately, this generator has other capacities that can be exploited, even if this is not what they were designed for. This generator has 12 channels, as we said before. Each channel corresponds to a simulated version of a GPS satellite signal. The generator gives the possibility to duplicate the same signal on several other channels (generally for multipath studies). Once the signal is duplicated, several parameters can be changed on the replicated channel, but without affecting the original. Amongst the possible settings, we can change the receiving delay between the original signal and the replicated one. Thus, for a simulated position, we can select 6 satellites giving the best dilution of precision and then replicate them on the 6 others channels. We can adapt the relative delays of these 6 replica signals to the 6 originals to make them correspond to another position (the fake position provided by the spoofer). We also added the same delay on each replica signal. This delay corresponded to a simulated distance between the spoofer and the receiver antennas. This “distance” simulated the propagation delay between the antennas plus the difference of the synchronization of the spoofer clock on the real constellation reference time. Figure 15 shows the setup for this experimentation.

The Simulated Propagation Spoofer to Receiver Delay (SPSRD) was 60 m. The following script was applied during the simulation:

- At t=0 s: start of the simulation, 6 true constellation channels on, 6 spoofer constellation channels off;
- At t=85 s: 6 spoofer constellation channels on with 10 dB of power more than the 6 true constellation channels.

**Figure 16:**
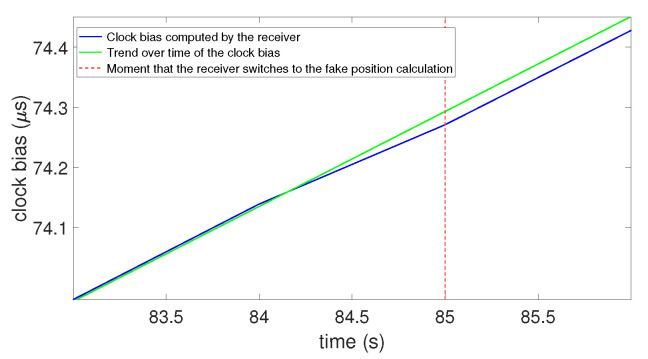
Clock bias with two perfectly synchronized constellations.

This corresponds to the “Strong attack”, but with a synchronized constellation. Figure 16 gives the results of this experimentation.

In Figure 16, the blue line is the clock bias computed by the receiver, the green dashed line is a line marking the trend over time of the clock bias, and the vertical red dashed line shows the moment that the receiver switches to the fake position calculation. This figure shows that, similar to the model, there was a jump of the value in the clock bias when the receiver locked onto the spoofing signals. Nevertheless, we can highlight that the amplitudes of the clock bias jump conformed to what was expected in the model: for 60 m (0.2 μs) of induced delay for a result averaging 0.22 μs. This confirmed the findings highlighted by our model (cf. Section 3.4). This experimentation and the way it worked led us to the ability to characterize the capacity of a receiver to detect a spoofing attack with the clock bias. We see this in the next session.

### 5.2. Determination of the Receiver Operating Characteristic of Clock Bias Jump Detection

It is fairly classical to determine the ROC curves for a detection system. We explain in this section how to use the previous experimental setup to determine these curves:

1. **First step:** Determine the mean level of the C/N0 for which the curve will be determined.
2. **Second step:** Choose the minimum and maximum values of the interval of the SPSRD that will be studied. Generally, we took 0 μs for the first one, which is very in favor of the attacker. The second value depends on the maximum expected distance and/or clock difference between the spoofer constellation and the real one.
3. **Third step:** Carry out the same experimentation as in Section 5.1, changing the SPSRD, and gather the result after several tests according to the expected accuracy (each experimentation can be repeated a minimum of 10 times to 100 times).

**Figure 17:**
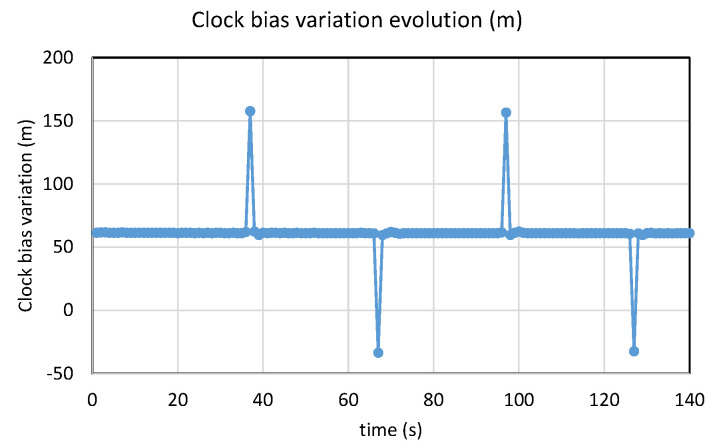
Example of evolution of clock bias variation, switching power.

As an example of the setting for the step, we can program the generator for several minutes. Every 30 s, we switched the maximum power from one constellation to the other. The strongest constellation will change every 30 s, and the jump can be observed in one sense or the other, as in Figure 17, and then counted. Note that, for convenience and the readability of the figure, we considered the variation of the clock bias obtained by differencing two successive samples, and not the clock bias directly.

The Probability of False Alarm (PFA) and the corresponding Probability of Detection (PD) were determined for each value of the threshold from 5 m to 0 m. Concretely, when the clock bias variation overtook the threshold and there was a jump, we counted one for the PD. If the values overtook the threshold without attack, we counted one for the PFA.

Repeating these experimentations several times for the different values of SPSRD, defined at Step 2, we obtained the ROC curve.

**Figure 18:**
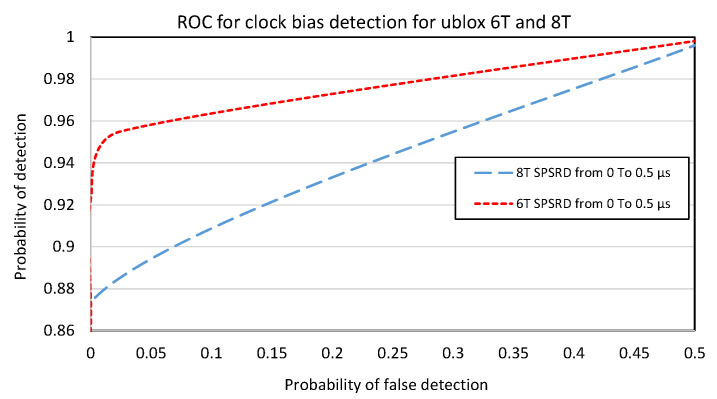
ROC curves for u-blox 6T and 8T for the SPSRD from 0 to 150 m.

We performed this for both receivers, and we obtained the results presented in Figure 18.

Note: The mean C/N0 is 36 dB-Hz for u-blox 6T and 44 dB-Hz for u-blox 8T (but the output signals from the generator are the same). It seems that the noise has no spectacular influence on the detecting capacity. We have curves that are close to the ideal detector, considering the simplicity of the considered detector. However, it is interesting to notice a paradox: considering this clock bias approach, the oldest generation of receiver (u-blox 6T) seems better than the most-recent (u-blox 8T). This is not clear from the figure, but with a threshold of 5 m, the probability of detection was 0.73 for the u-blox 8T and 0.91 for the u-blox 6T. Our ability to detect it depended only on the noise in the calculation of the clock bias. Here, we were obliged to use a result of the calculation of the Position, Velocity, and Time (PTV); we must thus take into account the behavior related to the positioning algorithm of the u-blox that we do not know. This is precisely what is characterized by these curves and differs between two versions of the receiver for the same manufacturer. A possible hypothesis is that the positioning of u-blox 6T is less sophisticated than the one of u-blox 8T, so more sensitive to jump. There was a smoothing effect, which was more important in u-blox 8T (this confirmed the previous experimentations). This was reinforced by the phenomenon that the curves did not highlight. For smaller values of the SPSRD (above 10 to 20 m), the u-blox 8T completely smoothed the jump. For larger values, it first smoothed the jump, but after a few seconds, it launched a hot start and recalculated the position. Thus, the jump was visible. We assumed that must be a solution integrity checking that detects a too large gap between the measures and the calculated position. It restarts the calculation.

Of course, all these remarks are valid for these two specific receivers only. However, with the tool and the method described in this section, this can be applied to any kind of commercial receiver for which the clock bias is available. This will provide an idea of the capacity to use this approach.

## 6. Discussion

Of course, the approach presented in the paper, which consisted of detecting the jump in the clock bias, is far from resolving all cases. The curves presented are valid for the experiment carried out, which, although it simulated very favorable conditions for the attacker (same constellations, sufficient signal strength), does not represent a receiver attacked in real conditions. Above all, there are other phenomena likely to cause jumps in the clock bias than the attack of a spoofer: change of constellation, appearance/disappearance of a satellite, masking, etc. However, the mathematical filters set up for the calculation of the clock bias by the receivers in question had a beneficial smoothing effect on these common phenomena.

However, what would happen with a particularly smart spoofer? If we take the example of the u-blox 8T, for the highest threshold (5 m), the SPSRD must be less than 150 m to fail the detection systematically. If we now consider a more sophisticated spoofer that synchronizes its signals with those of the constellation, with the ability to cancel the clock bias of its own oscillator, it must also have the ability to advance its own clock to cancel the effects of the distance between it and the receiver (reduce the SPSRD to 0 at the level of the spoofed receiver antenna). This is not impossible to do, but it is more complicated and restricts the “stealth” area of the spoofer to no more than 300 m in diameter (or maximum length); otherwise, the u-blox 8T receivers will be able to detect the attack. The conclusion was that, although the approach can be defeated, it still has the potential to work relatively well against a coherent spoofing attack of the second or third category described in Section 2.1.

## 7. Conclusions

In this paper, a model emulating a simple spoofing attack was developed. This model showed that even a well-designed spoofer causes disturbances to the behavior of the clock bias of the receiver. This translates into a jump of value when the receiver switches from the real signals to the spoofer signals. The model showed that the amplitude of this jump is related to the distance between the spoofing antenna and the target’s position. It is also related to the difference between the real constellation time (GNSS time) and the signal spoofer reference time. To validate this observation, spoofing attacks on a commercial GPS receiver were carried out with the use of GNSS signal simulators. Our experiments showed that, even on a real GPS receiver, the clock bias undergoes a leap of value when switching constellation. However, further investigations showed that the amplitude of the clock bias is not consistent, but is rather random, which makes it unpredictable. The measured amplitudes of the clock bias jumps during our experiments were mostly significant. From this, we developed and presented a method to characterize the ability of a receiver to detect a spoofing attack with clock bias monitoring. Therefore, based on that, a spoofing detection method based on the monitoring of the clock bias can be developed on a system using a COTS receiver, such as an UAV. For instance, a research team has developed an interesting method to protect an area from unintentional drones with a single signal spoofer [23]. They recognized that clock bias monitoring can undermine the method. Such a method does not require additional hardware or high computational resources, since the clock bias and the clock drift are already available on the GNSS receiver, which makes it a low-cost solution. The next steps could be to apply the described method of characterization to other commercial receivers than the ones presented here. The approach, limited to L1 GPS signals because of the limitation of the generators involved here, can be upgraded to other constellations and other signals with lower-cost solutions such as software-defined radio devices programmed as constellation generators. 

## Figures and Tables

**Table 1:** Mean leap in drift and bias and C/$N_{o}$ ratio.

Attack Type	U-Blox Receiver	Drift (μs/s)	Bias (μs)	C/$N_{o}$ (dBHz)
Acquisition	u-blox 6	0.016	1317.3	48–50
	u-blox 8	0.0165	498.1	48–50
Smooth	u-blox 6	0.0153	666.13	49–51
	u-blox 8	0.0163	774.53	49–51
Strong	u-blox 6	0.0118	776.32	50–52
	u-blox 8	0.0178	936.02	50–52
Jamming	u-blox 6	0.0133	393.4	41–43
	u-blox 8	0.018	986.2	41–43

## Abbreviations

The following abbreviations are used in this manuscript:

AGC	Automatic Gain Control
COST	Commercial-Off-The-Shelf
ECDF	Empirical Cumulative Distribution Function
ECEF	Earth-Centered Earth-Fixed
GLONASS	GLObal NAvigation Satellite System
GNSS	Global Navigation Satellite System
GPS	Global Positioning System
IMU	Inertial Measurement Units
PD	Power Distortion
RAIM	Receiver Autonomous Integrity Monitoring
SV	Space Vehicle
TCXO	Temperature-Compensated Crystal Oscillator
TOA	Time Of Arrival
UAV	Unmanned Aerial Vehicles
